# Examining how customers perceive community pharmacies based on Google maps reviews: Multivariable and sentiment analysis

**DOI:** 10.1016/j.rcsop.2024.100498

**Published:** 2024-08-23

**Authors:** Yahya Ali Laghbi, Mohammed Al Dhoayan

**Affiliations:** a Department of Health Informatics, CPHHI, King Saud Bin Abdulaziz University for Health Sciences, Riyadh, Saudi Arabia; b King Abdullah International Medical Research Center, Riyadh, Saudi Arabia

**Keywords:** Community pharmacies - Google maps, Sentiment analysis - consumer-generated content-exploratory data analysis

## Abstract

**Objective:**

This study aims to understand customer perceptions of community pharmacies utilizing publicly available data from Google Maps platform.

**Materials and methods:**

Python was used to scrape data with Google Maps APIs. As a result, 17,237 reviews were collected from 512 pharmacies distributed over Riyadh city, Saudi Arabia. Logistic regression was conducted to test the relationships between multiple variables and the given score. In addition, sentiment analysis using VADER (Valence Aware Dictionary for Sentiment Reasoning) model was conducted on written reviews, followed by cross-tabulation and chi-square tests.

**Results:**

The Logistic regression model implies that a unit increase in the Pharmacy score enhances the odds of attaining a higher score by approximately 3.734 times. The Mann–Whitney *U* test showed that a notable and statistically significant difference between “written reviews” and “unwritten reviews” (U = 39,928,072.5, *p* < 0.001). The Pearson chi-square test generated a value of 2991.315 with 8 degrees of freedom, leading to a *p* value of 0.000.

**Discussion:**

Our study found that the willingness of reviewers to write reviews depends on their perception. This study provides a descriptive analysis of conducted sentiment analysis using VADAR. The chi-square test indicates a significant relationship between rating scores and review sentiments.

**Conclusion:**

This study offers valuable findings on customer perception of community pharmacies using a new source of data.

## Introduction

Community pharmacies are healthcare facilities where pharmacists provide pharmaceutical services to the local community. These establishments play a vital role in providing medications, health advice, and other related services to the public. The organizational factors associated with community pharmacies, such as public relations, staffing, and service quality, can significantly impact the services provided and customer perception.^1^ Additionally, the culture within community pharmacy organizations can influence the services delivered, the pharmacy workforce, and overall business outcomes.^2^

lack of patient satisfaction and ignoring the patient's perspective when evaluating the quality of clinical treatment may cause medical therapy to fail and waste health care resources. When evaluating the effectiveness of clinical treatment, dissatisfaction and contempt for the patient's perspective may lead to failed medical therapy and the waste of health resources.^3^ El-Sharif et al.^4^ claim that a patient's expectations and past experiences influence how positively they feel about the service. It is vital to consider patients' views and satisfaction with the services provided by community pharmacies because it has been demonstrated that increasing patient satisfaction significantly improves patients' adherence to medications and, as a result, treatment outcomes.

Several interconnected elements affect how satisfied patients are with pharmacy services. These factors include the atmosphere at the pharmacy, the time spent waiting, the confidentiality of the consultation, the ability to communicate, the method of dispensing, and the availability and storage of medications. Therefore, it is essential to gauge patient satisfaction with the pharmacological care provided in community pharmacies.^5^

Satisfaction is a trustworthy indicator of the caliber of medical care. Low patient satisfaction levels may result in losing consumers since disgruntled patients may not be inclined to return. Still, high patient satisfaction levels demonstrate that community pharmacies are performing their tasks well. However, low satisfaction levels might encourage pharmacists to improve several patient care elements.^6^

Customer perception refers to how individuals perceive and evaluate the services, products, and overall experience provided by businesses, including community pharmacies. In the context of community pharmacies, customer perception plays a crucial role in influencing customer behavior, satisfaction, and loyalty. Understanding customer perception in community pharmacies involves assessing factors such as service quality, cost, value, satisfaction, and loyalty.^7^ Customer perception is shaped by various elements, including service quality, customer satisfaction, and expectations, which are essential components in determining customer behavior and loyalty toward community pharmacies.^8^

The pharmacist must ensure public acceptance while the profession attempts to improve its clinical function. Numerous reports, research, and polls worldwide have assessed how patients feel about and are satisfied with community pharmacies' services. People in industrialized nations such as the United States, the United Kingdom, and Canada have favorable views of community pharmacy services.^9^ Certainty, empathy, and responsiveness have a strong correlation with customer satisfaction, according to Cavana et al.^10^ research.

### Google maps platform

Google Maps reviews (or other platforms) have been performed and used to evaluate or analyze customer behavior as one method of evaluating consumer behavior. Sharing ideas and experiences about the caliber and usability of products and services has long benefited consumers. Ratings and reviews are more accessible, familiar, and important than ever because of the growth of the internet, e-commerce, and online platforms. Google Local Guide is a feature of Google Maps platform through the act of sharing reviews, photos, videos, and experiences of visited places with other users, local guides earn points and level up through contributions.^11^

To help them make buying decisions, millions of consumers create and rely on the ratings and reviews left by other customers on Google Maps. Reading online customer ratings and reviews can help prospective customers evaluate a seller's past performance and do so more quickly and with more excellent knowledge. They produce initial sales, client satisfaction, repeat business, profit, and shareholder value.^12^ According to a 2013 US study, 96 % of businesses perceive customer reviews from Google Maps ratings as an effective method to increase conversion.^13^

Sentiment analysis is the study of people's opinions, emotions, perception toward things such as goods and services. Sentiment words, also known as opinion words, are the most significant of sentiments where those words are commonly used to express positive or negative sentiments.^14^ Sentiment analysis using a text mining technique in the feedback review column is essential to ascertain end-user perceptions of the conditions of the goods and services provided.^15^ Vu, et al.^16^ researched examining visitor eating preferences using text processing techniques and online restaurant reviews to close the gap. An extensive data collection of more than 40,000 restaurant reviews submitted by patrons of 2265 different businesses was utilized in a case study on foreign tourists visiting Australia to demonstrate the value of the google review system. The recommended methods might help researchers get a complete insight into visitors' dietary preferences.

In contrast to the healthcare sector, Google Maps reviews has been used effectively in other service industries such as tourism and hospitality. Chittiprolu et al.^17^ investigated the opinions of hotel staff members about the management style each hotel employed. Aakash and Gupta^18^ investigated Google maps with big data analysis and text mining and discovered that it provided more insight into hotel performance and client satisfaction.

The theoretical framework underpinning sentiment analysis and logistic regression in the context of pharmacy services involves analyzing customer sentiment and behavior toward pharmacy services. Sentiment analysis aims to understand and extract subjective information from customer reviews, feedback, and social media data to gauge customer satisfaction and perception.^19^ Logistic regression, on the other hand, is a statistical method used to model the relationship between various factors and a binary outcome, such as customer satisfaction or loyalty in the context of pharmacy services.^8^

This study aims to explore customer perception toward community pharmacies through multivariable analysis based on data collected from Google Maps reviews (review time, user review count, user local guide, review text, review likes, pharmacy score, and the given score), and to conduct sentiment analysis to understand the relationship between review's sentiment and the given score in Google Maps reviews.

## Methods

### Data collection

**Fig. 1:**
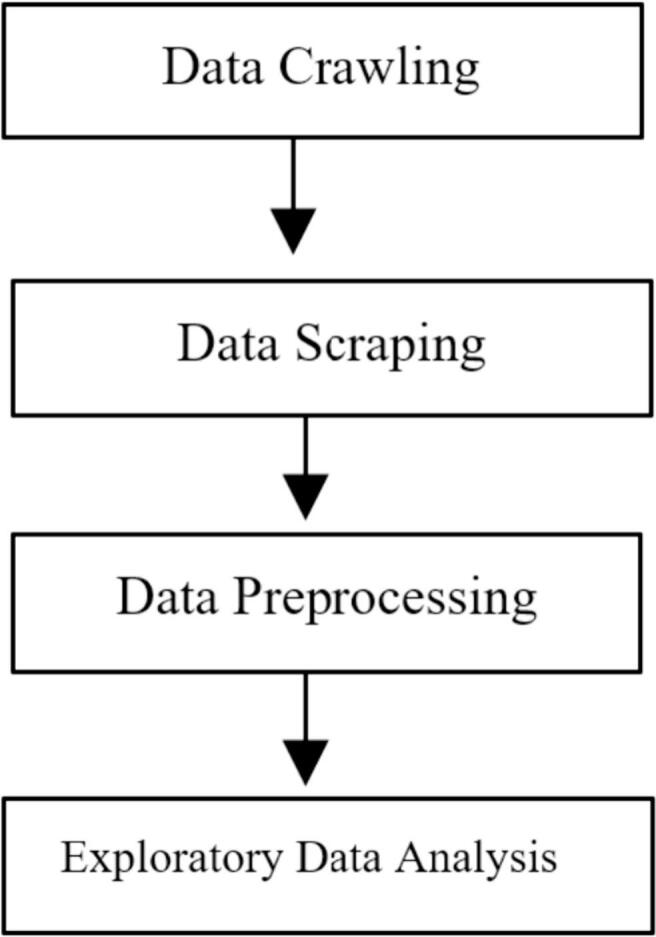
Data collection and analysis process.

Data for this study were collected from publicly available data on the Google Map platform. The data collection process began by crawling all available URLs of community pharmacies listed in Google Maps within the city of Riyadh, Saudi Arabia. To ensure representative sampling, Riyadh city was categorized into four regions: north, south, east, and west, based on Riyadh region municipality.^20^ Manual collection of URLs was then conducted quarterly by inspecting Google Maps listed pharmacies in each neighborhood, ensuring comprehensive coverage. The Python programming language was utilized for scraping the data using Google Maps APIs (Application Programming Interface). This systematic approach contributed to the robustness of our dataset, ensuring efficient and comprehensive retrieval. The entire collection process spanned from Jan 1 to 10, 2023, with a retrospective inclusion of all reviews before Jan 1, 2023. The scraped data for each review encompassed review time, user review count, user local guide, review text, review likes, pharmacy score, and score, with each review assigned a unique ID and each reviewer identified by a customer identification (CID). Additionally, each pharmacy was associated with a unique place ID. The subsequent step involved data cleaning, removing any duplicate, incorrect, or inconsistent entries to ensure the quality of the dataset. This meticulous process resulted in 17,237 reviews collected from 512 pharmacies distributed across Riyadh city, as illustrated in Fig. 1. The combination of categorization, manual collection, and advanced programming contributed to a comprehensive and reliable dataset for subsequent analysis.

### Statistical analysis

#### Logistic regression

The test conducted offers insights from a logistic regression model concerning the ‘score’ as the dependent variable. Four predictor variables linked with each review were used: total likes, pharmacy total reviews, user review count, local guide status, and pharmacy score. Logistic regression is well-suited for modeling the probability of a categorical outcome, providing valuable insights into pharmacy scores. Considering ‘score’ as an ordinal variable aligns with the nature of our dependent variable. The ordinal logistic regression was used here, as we considered score as an ordinal variable. The test was conducted mainly to determine if the dependent variable ‘score’ could be predicted based on four predictor variables.

#### Comparison of written reviews and unwritten reviews

The main aim of this test was to identify any significant differences in perceptions about pharmacy services between two distinct groups of participants, categorized by their involvement in writing reviews. Two groups were compared based on the given score by customers. Categorizing users based on their review-writing behavior enables us to explore potential variations in their evaluations, contributing to a nuanced understanding of customer perspectives.

### Text analysis

#### Sentiment and cross-tabulation analysis

Sentiment analysis, a method exploring people's opinions and emotions toward various subjects, was applied in our study specifically to written reviews, refining the dataset for analysis. Out of the 17,237 reviews, 10,676 were identified as written reviews, and a meticulous data cleaning process was executed on this subset to eliminate meaningless text and reviews containing only emojis. This process resulted in 8804 valid reviews selected for sentiment analysis using the VADER (Valence Aware Dictionary for Sentiment Reasoning) model. Renowned for its efficiency and accuracy, VADER is a lexicon and rule-based sentiment analysis tool capable of detecting sentence polarity (positive, neutral, or negative) and intensity of sentiment. Importantly, VADER has demonstrated its effectiveness, even surpassing individual human raters in achieving high-quality results.^21^ This analysis provides qualitative insights into customer sentiments, enhancing our understanding of the emotional tone expressed in reviews, while the reliability of the VADER model ensures robust sentiment analysis outcomes.

Cross-tabulation analysis was conducted based on the results of the sentiment analysis, utilizing subsequent chi-square tests on a dataset to explore the potential connection between two variables: “Score” and “Sentiments.” The focus on cross-tabulation allowed for the exploration of associative patterns between score categories and sentiment, contributing to a more comprehensive understanding of the factors influencing customer ratings. This analytical approach provides valuable insights into the interplay between sentiment expressed in reviews and the corresponding scores, aiding in the identification of nuanced relationships that contribute to a more thorough interpretation of customer perceptions and preferences.

## Results

### Frequency

Table 1 provides the complete details of the score and user guide frequencies of the data used in this study.

**Table 1:** Frequency.

Items	Frequency (*N* = 17,237)	(%)
***Score (Star rating)***		
1	2992	17.4
2	650	3.8
3	1808	10.5
4	2623	15.2
5	9164	53.2
***Local guide status***		
No	6753	39.2
Yes	10,484	60.8

### Descriptive statistics

Table 2 shows the descriptive statistics, i.e., mean std. deviation minimum, and maximum of all the continuous variables.

**Table 2:** Descriptive statistics.

	N	Minimum	Maximum	Mean	Std. Deviation
Pharmacy score	17,237	1.8	5	3.831	0.415
Total likes	17,237	0	81	0.400	1.264
Total reviews	17,237	4	249	57.740	45.858
User reviews count	17,237	0	2871	108.590	197.726

### Ordinal logistic regression

The p value (< 0.001) (see Table 3) associated with the chi-squared statistic indicates that the ordinal regression model has a significant fit to the data, implying that observed differences between predicted and actual outcomes are not due to random chance alone.

**Table 3:** Model Fit Measures.

				Overall Model Test					
Model		R^2^_McF_		χ^2^		df		p	
1		0.0309		1369		5		< 0.001	

The dependent variable ‘score’ is rated in the following order: 1 | 2 | 3| 4 | 5.

Table 4 provides insights from a logistic regression model with ‘score’ as the dependent variable. Each predictor variable's impact on achieving different score categories is outlined through their respective coefficients and statistics. Notably, the Pharmacy score exhibits an estimated coefficient of 1.31747, signifying an Odds Ratio of 3.734 (χ^2^ = 1159.68, *p* < 0.001). This implies that a unit increase in the Pharmacy score enhances the odds of attaining a higher score by approximately 3.734 times. Conversely, ‘Total likes’ display a coefficient of −0.12962, corresponding to an Odds Ratio of 0.878 (χ^2^ = 82.70, p < 0.001), indicating a decrease in the odds of achieving a higher score as Total likes increase. Similarly, the Total reviews coefficient is −0.00103, yielding an Odds Ratio of 0.999 (χ^2^ = 10.16, *p* = 0.001), suggesting minimal odds change for a unit increase in Total reviews. User reviews count shows an Odds Ratio of 1.000, with a negligible coefficient of −2.14e-4 (χ^2^ = 8.66, *p* = 0.003), implying limited influence on higher score odds. Lastly, for the variable local guide (Yes – No), the coefficient is 0.00628, reflecting an Odds Ratio of 1.006 (χ^2^ = 0.04, *p* = 0.846), indicating minimal impact on the odds of achieving higher scores based on local guide status.

**Table 4:** Parameter Estimate and Model Coefficients (Outcome variable: score).

Predictor	Estimate	SE	Z	χ^2^	p	Odds ratio
Pharmacy score	1.31747	0.0387	34.054	1159.68	< 0.001	3.734
Total likes	−0.12962	0.0143	−9.094	82.7	< 0.001	0.878
Total reviews	−0.00103	0.000324	−3.187	10.16	0.001	0.999
User reviews count	−2.14e−4	7.27E-05	−2.944	8.66	0.003	1.000
Local guide:						
Yes – No	0.00628	0.0323	0.195	0.04	0.846	1.006

No is the reference category.

### Comparison of written reviews and unwritten reviews

In order to identify any significant differences in perceptions about pharmacy services between two distinct groups of participants, categorized by their involvement in writing reviews. The “written reviews” group encompassed 10,676 respondents who voluntarily contributed reviews, indicative of their positive sentiment toward the services. Conversely, the “unwritten reviews” group comprised 6561 respondents who had refrained from writing reviews, potentially suggesting a comparatively less favorable opinion (see Table 1). The “written reviews” group exhibited a mean rank of 8159.52, whereas the “unwritten reviews” group had a higher mean rank of 9366.67 (see Table 5). The Mann–Whitney *U* test showed that a notable and statistically significant difference emerged between these groups (U = 39,928,072.5, *p* < 0.001). Notably, the “written reviews” group exhibited a lower mean rank (8159.52) than the “unwritten reviews” group (9366.67). Crucially, the calculated effect size, represented by the standardized test statistic of 16.876, corresponds to a magnitude of approximately 0.128 (see Table 5).

**Table 5:** Independent-samples Mann-Whitney *U* test summary.

Review Type	Total N	Mann-Whitney U	Wilcoxon W	Test Statistic	Standard Error	Standardized Test Statistic (z)	Asymptotic Sig. (2-sided test)	Effect Size (z / √N)
Written Reviews	8600	19,741,231	26,464,796	19,741,231	290,684.7	16.876	0.000	0.128
Unwritten Reviews	8637	20,184,540	26,909,440	20,184,540	290,684.7	16.876	0.000	

### Text analysis

#### Sentiment analysis

**Fig. 2:**
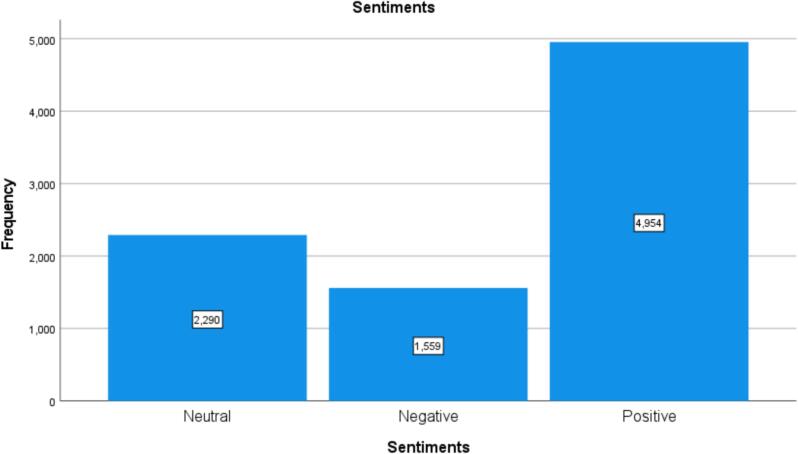
Graphical representation of Sentiments.

**Fig. 3:**
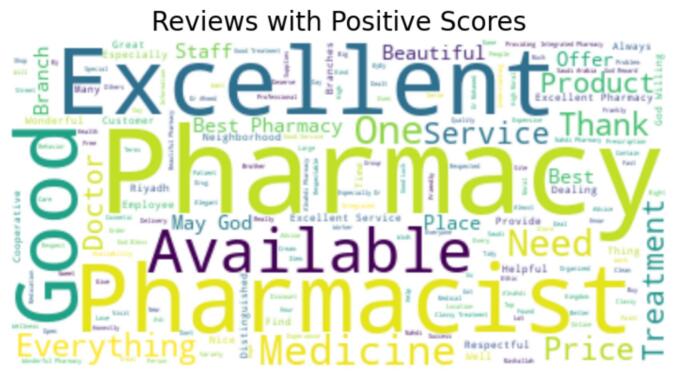
Word Cloud of reviews with positive Scores.

**Fig. 4:**
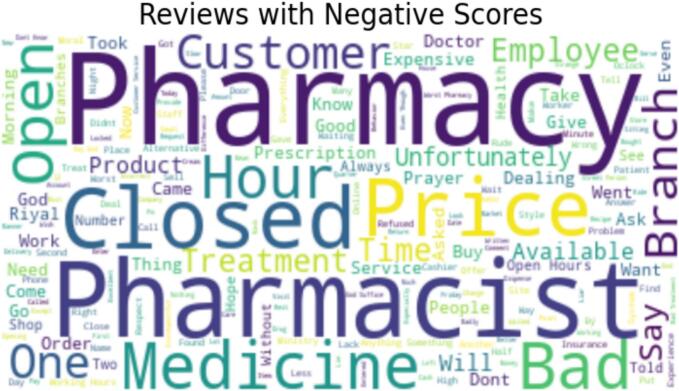
Word Cloud of reviews with negative Scores.

This section presents sentiment analysis to examine customers' subjective opinions after visiting pharmacies. First, we cleaned the data for the 10,676 written reviews to remove meaningless text or reviews that only contained emojis. As a result, 8084 reviews were eligible for sentiment analysis. To demonstrate, we used VADER (Valence Aware Dictionary for Sentiment Inference), which is a Natural Language Toolkit (NLTK) module that provides sentiment scores from 1 to −1 based on the word used in the review.^22^ VADER is a rule-based sentiment analyzer where reviews are generally classified based on sentiment scores to positive sentiment (score ≥ 0.05), neutral sentiment (score > −0.05) and (score < 0.05), and negative sentiment (score ≤ −0.05) (see Fig. 2). Additionally, we used the Word cloud library in Python to identify the most common words in positive and negative reviews (see Fig. 3, Fig. 4).

## Graphical representation of Sentiments

### Cross-tabulation analysis

The following tables showcase the outcomes of cross-tabulation analysis and subsequent chi-square tests performed on a dataset to explore the potential connection between two variables: “Score” and “Sentiments.” The cross-tabulation table reveals the distribution of reviews categorized by different sentiment levels (“Neutral,” “Negative,” and “Positive”) within each distinct “Score” category. For instance, within the “1” score category, there were 675 reviews categorized as “Neutral,” 1145 as “Negative,” and 483 as “Positive,” resulting in a cumulative total of 2303 reviews. Similar counts were calculated for the other score categories (see Table 6).

**Table 6:** Score* Sentiments Crosstabulation.

		Sentiments			
		Neutral	Negative	Positive	Total
Score	hated it	675	1145	483	2303
	disliked it	143	109	114	366
	it was okay	297	117	414	828
	liked it	342	80	760	1182
	loved it	833	108	3183	4124
Total		2290	1559	4954	8803

Chi-square tests, which are utilized to determine the statistical significance of the association between the two variables, produced a variety of metrics. The Pearson chi-square test generated a value of 2991.315 with 8 degrees of freedom, leading to a *p* value of 0.000. Given that all *p* values are strikingly low, close to zero, it implies a noteworthy connection between “Score” and “Sentiments.” This suggests that the distribution of sentiments does not occur randomly across various score categories, thereby implying a substantial correlation between the assigned rating scores and the sentiments expressed in the reviews. Furthermore, it is important to highlight that all expected counts for the chi-square tests exceeded 5, ensuring the statistical reliability of the results.

## Discussion

This study is the first of its kind in the pharmaceutical sector, where Google Maps reviews are used as a data source to understand customer behavior and perceptions of community pharmacies instead of conventional data collection methods. The adoption of Google Maps in health care research is very slow, where it has been adopted well in other sectors, such as tourism, hospitality, and restaurants.^23^, ^24^, ^25^

Our study found a weak positive correlation between pharmacy score and given score, and between pharmacy score and pharmacy total reviews. These findings are consistent with a study on restaurant evaluations using online reviews, which showed that average rating and number of reviews influence how consumers evaluate a restaurant.^26^ This indicates that online reviews are a valuable tool for understanding customer perceptions across different industries.

Our study reveals that the willingness of reviewers to write reviews depends on their perception and establishes a significant link between writing reviews and positive perceptions of pharmacy services, emphasizing the potential role of reviews as indicators of satisfaction and contentment. The comparison of written reviews against no written reviews suggests that respondents who opted to write reviews were inclined to hold more favorable perceptions of pharmacy services compared to their counterparts who did not engage in review writing. This difference has been studied from a different perspective; a study conducted for public libraries found that the average length of written reviews varied inversely according to the given score.^27^

This study provides a descriptive analysis of conducted sentiment analysis using VADER. Moreover, word clouds visually represent and compare categories of positive and negative reviews, reflecting the most frequently used words in each category. Outcomes of the chi-square tests provide substantial evidence to support the notion of a significant relationship between the given rating scores and the sentiments conveyed in the reviews. This insight can be of immense value in comprehending customer perspectives and viewpoints, thereby enabling community pharmacies to tailor their strategies to augment customer contentment and refine overall sentiment. This study has confirmed the correlation between sentiment and given score.

The results of the logistic regression analysis also support the findings of pharmacy score as an influential factor that contributes to higher customer satisfaction. The odds ratios reveal that pharmacy scores are positively and significantly related to the overall scores, meaning that higher scores in pharmacy increase the chances of getting better scores in the other domains. On the other hand, the negative signs of the total likes and total reviews imply that these factors may not necessarily help in achieving higher satisfaction scores, which further explains the intricacies of customers' perception.

### Practical implications

Therefore, this study reveals that community pharmacies can use online reviews as a source of information on customers' perceptions and satisfaction. Pharmacies should therefore constantly analyze the reviews they receive so that they can see what specific issues customers have and how they can address them. For instance, the aspects that are considered to be negative by the customers, for instance, the time spent waiting or the attitude of the staff of the pharmacy, can be worked on to increase the satisfaction of the customers. In addition, analyzing the sentiment of the comments left by customers will allow pharmacies to adjust their approach to communication and service provision to the needs and expectations of the customers.

### Recommendations

#### Enhance online presence

Pharmacies should actively manage their online presence by responding to reviews and engaging with customers. This can help build trust and demonstrate a commitment to customer service.

#### Use review data

Regularly analyze review data to identify trends and areas for improvement. Implementing changes based on customer feedback can lead to better service quality and higher satisfaction levels.

#### Train staff

Provide training for pharmacy staff to improve customer interactions, focusing on areas highlighted in negative reviews. Effective communication and empathy can significantly enhance customer perceptions.

#### Encourage reviews

Encourage customers to leave reviews. This can help to provide a more accurate representation of the pharmacy's services.

### Broader implications

The broader implications of this study extend to the healthcare sector, emphasizing the need for healthcare providers to engage with digital feedback mechanisms actively. This approach can be instrumental in continuously improving service quality and patient satisfaction. Additionally, the findings suggest that future research should explore the integration of online reviews from multiple platforms and across different cities to provide a more comprehensive understanding of customer perceptions in various contexts.

### Future research directions

Future research is required to investigate the characteristics concerning customer satisfaction and develop a predictive model to estimate the given score using sentiment reviews. Extending the research to cover more platforms and cities and making a distinction between the chain and independent pharmacies would give a better picture of the customer sentiments in the community pharmacy segment. Furthermore, analyzing temporal aspects of the review patterns and their relation to the changes in services may provide more profound understanding of the efficiency of the applied measures.

## Funding

None.

## CRediT authorship contribution statement

**Yahya Ali Laghbi:** Writing – review & editing, Writing – original draft, Visualization, Project administration, Methodology, Data curation, Conceptualization. **Mohammed Al Dhoayan:** Writing – review & editing, Supervision, Software, Resources, Methodology, Investigation, Conceptualization.

## Declaration of competing interest

The authors declare that they have no known competing financial interests or personal relationships that could have appeared to influence the work reported in this paper.
